# Progress and Prospects of Anti-HBV Gene Therapy Development

**DOI:** 10.3390/ijms160817589

**Published:** 2015-07-31

**Authors:** Mohube B. Maepa, Ilke Roelofse, Abdullah Ely, Patrick Arbuthnot

**Affiliations:** Wits/SAMRC Antiviral Gene Therapy Research Unit, School of Pathology, Health Sciences Faculty, University of the Witwatersrand, Wits 2050, South Africa; E-Mails: Betty.Maepa@wits.ac.za (M.B.M.); Ilke.Roelofse@wits.ac.za (I.R.); Abdullah.Ely@wits.ac.za (A.E.)

**Keywords:** HBV, gene therapy, RNAi, gene editing, antisense oligonucleotides, ribozymes

## Abstract

Despite the availability of an effective vaccine against hepatitis B virus (HBV), chronic infection with the virus remains a major global health concern. Current drugs against HBV infection are limited by emergence of resistance and rarely achieve complete viral clearance. This has prompted vigorous research on developing better drugs against chronic HBV infection. Advances in understanding the life cycle of HBV and improvements in gene-disabling technologies have been impressive. This has led to development of better HBV infection models and discovery of new drug candidates. Ideally, a regimen against chronic HBV infection should completely eliminate all viral replicative intermediates, especially covalently closed circular DNA (cccDNA). For the past few decades, nucleic acid-based therapy has emerged as an attractive alternative that may result in complete clearance of HBV in infected patients. Several genetic anti-HBV strategies have been developed. The most studied approaches include the use of antisense oligonucleotides, ribozymes, RNA interference effectors and gene editing tools. This review will summarize recent developments and progress made in the use of gene therapy against HBV.

## 1. Introduction

World-wide, over 240 million people are chronically infected with hepatitis B virus (HBV), and approximately one million people die from the infection every year (reviewed in [1]). HBV can be transmitted sexually or percutaneously, however, transmission during early life poses a high risk of developing persistent infection. Viral persistence may predispose patients to serious diseases, such as cirrhosis and hepatocellular carcinoma [2]. The virion of HBV is a small, enveloped, hepatotropic particle that contains an incompletely closed double-stranded DNA genome of 3.2 kb. Its genome encodes four partially overlapping open reading frames (ORFs). *Pre*-*core*/*core* (*pre*-*C*/*C*) ORF encodes nucleocapsid (Core/C) and the secreted pre-core protein, which is processed to produce hepatitis B e antigen (HBeAg). The *polymerase* (*pol*) ORF encodes the viral polymerase comprised of the reverse transcriptase (RT), RNase H and terminal protein domains. The *pre*-*surface*/*surface* (*pre*-*S*/*S*) ORF encodes pre-S1, pre-S2 and Surface (S) proteins. The *X* ORF encodes the regulatory X protein (reviewed in [3,4]). Drugs currently approved for HBV infection treatment are the immune modulators (conventional interferon (IFN)-α and PEGylated IFN-α), which stimulate the immune system to clear infected hepatocytes, and nucleosides or nucleotide analogs (lamivudine, adefovir, entecavir, telbivudine, dipivoxil and tenofovir), which inhibit reverse transcription. These drugs efficiently reduce viral replication and delay complications of chronic HBV infection, but complete clearance of the virus is rarely achieved [5,6]. Also, long-term use of therapy may be associated with emergence of resistant viral strains and toxicity. These factors have prompted extensive research aimed at understanding the biology of HBV, with the goal of identifying new drug targets.

**Figure 1:**
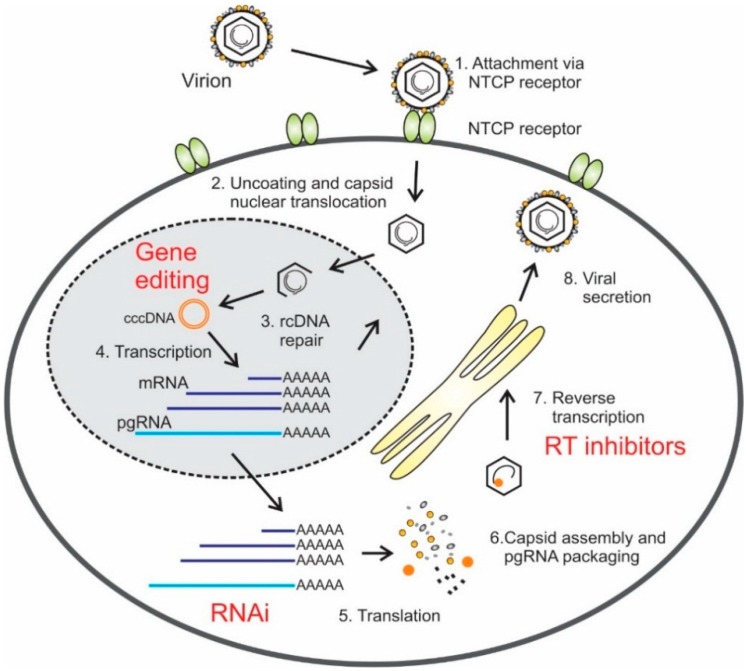
Diagram of hepatitis B virus (HBV) replication cycle. Attachment to the sodium taurocholate co-transporting polypeptide (NTCP) receptor, and possibly other receptors too, is the initiating event of infection (**1**); After uncoating and nuclear translocation of the capsid, relaxed circular DNA (rcDNA) is delivered to the nucleus (**2**); rcDNA is then repaired to form covalently closed circular DNA (cccDNA) (**3**); which is the template for transcription of viral RNA (**4**); Viral mRNA is translated (**5**); The pre-genomic RNA (pgRNA) is then packaged into capsid particles together with the viral Pol (**6**); The pgRNA is reverse transcribed in the nucleocapsid (**7**); And the viral particles are secreted via the endoplasmic reticulum (**8**). Sites of action of licensed and potentially therapeutic agents are indicated in red text. Viral cccDNA may be disabled by methods that employ gene editing. Exogenous activators of the RNA interference (RNAi) pathway may be employed to inactivate viral RNA. Nucleoside and nucleotide analogues, which are currently licensed drugs, may be used to inhibit reverse transcription of pgRNA.

The viral life cycle has a number of steps that are potential targets for antiviral drugs [7]. Infection is initiated by low affinity attachment of the virus to host surface heparan sulfate proteoglycans (HSP) [8].This is followed by high affinity attachment through interaction of viral pre-S1 with the host sodium taurocholate co-transporting polypeptide (NTCP) [9]. Internalization occurs by endocytosis or direct fusion of the plasma membrane with the viral envelope. Viral uncoating in the cytoplasm and nucleocapsid transport to the nucleus is followed by the repair of viral relaxed circular DNA (rcDNA) by host and viral machinery. This forms episomal covalently closed circular DNA (cccDNA), which is important for HBV persistence. The cccDNA functions as a template for the transcription of and the pre-genomic RNA (pgRNA), the *pre*-*C*/*C* mRNA, and the sub-genomic *surface* (*pre*-*S1* and *pre*-*S2*/*S*) and *X* mRNAs. Following nuclear export, the pgRNA may serve as a template for translation of the viral polymerase and capsid proteins or be encapsidated. Within the nucleocapsid, the pgRNA is reverse transcribed to form the viral negative DNA strand, which then serves as a template for plus strand synthesis during the generation of rcDNA. The nucleocapsid may either be enveloped and released via the endoplasmic reticulum (ER) or translocated to the nucleus for further cccDNA synthesis. The *pre*-*C*/*C* mRNA serves as template for the translation of the pre-core/core protein and the sub-genomic mRNAs are used for synthesis of the X-protein and three envelope proteins [5,7] (Figure 1).

Most current drugs target the reverse transcription stage of the HBV replication cycle. However, newer anti-HBV drugs targeting other stages are under development [10,11,12,13]. The recent discovery of the sodium taurocholate co-transporting polypeptide (NTCP) receptor as the viral entry receptor has been an important milestone in HBV biology [9].This multiple transmembrane transporter is predominantly expressed in the liver. Although several studies have suggested that unknown additional host factors are important for HBV entry, NTCP is currently the main known viral receptor [9,14,15]. Characterization of NTCP has facilitated development of better cell culture models of HBV infection and is also a new target for drug development. HBV entry inhibitors have thus recently gained significant attention, and several NTCP inhibitors are in clinical development (reviewed in [16]). Newer drugs targeting encapsidation (e.g., phenylpropenamide derivatives) are also under development [12].

**Figure 2:**
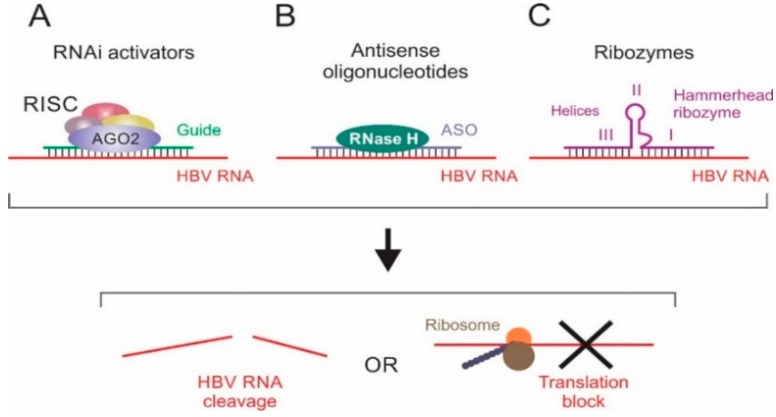
Gene therapy strategies targeting HBV RNA. (**A**) Expressed or synthetic activators are incorporated into the RNA-Induced Silencing Complex (RISC) to redirect the RNAi pathway to silence viral target sequences; (**B**) Antisense oligonucleotides (ASOs) suppress gene expression by binding to target RNA through classical Watson–Crick base pairing to block translation or induce RNase H-mediated RNA cleavage; (**C**) Ribozymes do not rely on the host machinery for cleavage, but possess an enzymatic domain (Helix II in hammerhead ribozymes) that cleaves the target RNA following sequence specific binding of the RNA binding domains (Helix I and Helix III). These strategies result in HBV RNA degradation or suppression of viral protein translation.

**Figure 3:**
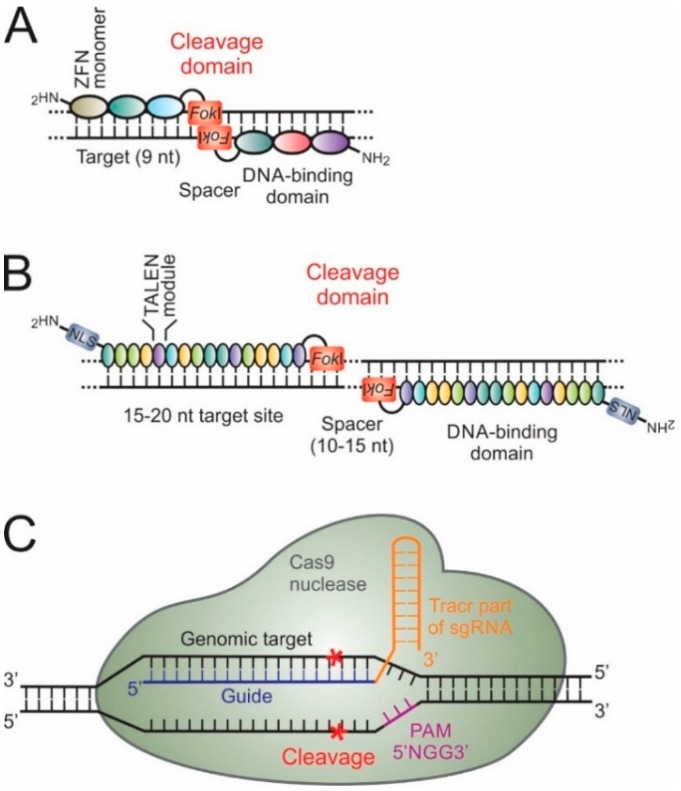
Engineered nucleases used in genome and epigenome editing. (**A**) Zinc finger nucleases (ZFNs) containing three zinc finger modules recognize a nine-nucleotide target DNA sequence on the sense and antisense strands. *Fok*I dimerization effects cleavage of both strands at the target site situated in the 5–7 nucleotide spacer region or cleavage domain; (**B**) Transcription activator-like effector nucleases (TALENs) comprise approximately 16 modules for each of the left and right subunits. Each module contains 33–35 amino acids that bind a single nucleotide at the repeat variable diresidue (RVD) at amino acids 12 and 13. Much like ZFNs, the *Fok*I nuclease domain must dimerize to cleave each of the strands of the duplex DNA; (**C**) Clustered regulatory interspaced short palindromic repeats (CRISPR) and CRISPR associated (Cas) proteins system with single guide RNA (sgRNA) comprising a combination of naturally occurring CRISPR RNA (crRNA) and transactivating CRISPR RNA (tracrRNA). The sgRNA guides the Cas9 endonuclease by binding genomic DNA with the aid of an obligate upstream protospacer adjacent motif (PAM) sequence to effect site-specific cleavage. nt: nucleotide. Red asterisks: cleavage site.

Treatment strategies using gene therapy have emerged as promising ways of countering viral infections. Various technologies have been vigorously pursued to develop treatment strategies for chronic HBV infection. Several methods have been developed to inhibit function of viral and host dependency factors [7,17]. Most nucleic acid-based strategies against HBV have employed the use of RNA interference (RNAi) effectors, gene editors, antisense oligonucleotides (ASOs) and ribonucleic acid enzymes (ribozymes) (Figure 1). RNAi effectors reprogram the natural RNAi pathway to target viral sequences and cause mRNA degradation or translational suppression (reviewed in [18]). ASOs are artificially synthesized and suppress gene expression by target hybridization and induction of RNase H-mediated cleavage of the viral mRNA [19]. Ribozymes are derived from naturally occurring RNAs that possess an antisense sequence that binds and enzymatically disables the target (Figure 2) (reviewed in [20,21]). A limitation of ASOs, artificial ribozymes and RNAi-based gene silencing is that eradication of HBV infection seems unlikely using these candidate drugs. By targeting and editing the viral cccDNA, designer nucleases are currently the most attractive agents for permanently inhibiting HBV replication. Zinc finger nucleases (ZFNs) and transcription activator-like effector nucleases (TALENs) have been successfully used to edit HBV cccDNA [22,23,24,25,26]. Recently discovered clustered regulatory interspaced short palindromic repeats (CRISPR) and CRISPR associated (Cas) protein endonucleases, which utilize RNA to guide binding to target DNA, have also emerged as potentially useful for cccDNA inactivation (Figure 3) [27,28,29].

Development of gene therapy against HBV has advanced significantly in the last decade. However, there are challenges that need to be overcome before anti-HBV gene therapy can enter the clinic. These include limiting toxicity, preventing emergence of viral resistance, ensuring specificity, a prolonged therapeutic effect and hepatocyte-targeted delivery. There has been significant progress in overcoming these obstacles, and some of the advances are discussed in this review.

## 2. Antisense Oligonucleotides

Antisense oligonucleotides are short, synthetic, nucleic acid fragments that specifically bind to the target RNA sequences to form a DNA:RNA hybrid (for antisense DNA) or RNA:RNA duplex (for antisense RNA). This may block reverse transcription or translation by ribosomal blockage or induce RNase H-mediated RNA cleavage of RNA:DNA hybrids (Figure 2) [19,30]. Since the first application of ASOs against HBV in 1990 [31], several studies have further demonstrated the potential of ASOs in gene therapy. Studies have reported efficient ASOs targeting *surface*, *pre-S1*, *pol*, *X* and *core* genes [31,32,33,34,35,36]. ASOs targeting non-coding sequences, such as the packaging and polyadenylation signals, are also effective against HBV replication [31,37,38]. Despite the encouraging data, ASOs against HBV have not yet progressed to clinical development. This is mainly as a result of challenges that include instability, inefficient intracellular delivery, inadequate affinity to targets and toxicities associated with ASOs. However, the approval of fomivirsen, an ASO for treatment of retinitis caused by cytomegalovirus, illustrates the potential of ASOs as antiviral agents [39].

Several groups have shown that ASOs can easily be modified at the base, phosphodiester groups and the sugar to increase stability, solubility, specificity and affinity for targets. The phosphate backbone is most commonly targeted for modification by replacing one oxygen atom with a sulfur atom (phosphorothioates) or methyl group (methylphosphonates) [40,41]. Antisense locked nucleic acids (LNAs), where the ribose ring is locked by connecting the 2′O atom and the 4′C atom of the oligonucleotide, have been used successfully against HBV with no evidence of toxicity [36,42]. Phosphorodiamidemorpholino oligomers (PMOs), which are DNA-like ASOs with the deoxyriboses substituted by six-membered morpholino rings and phosphodiester linkage replaced with uncharged phosphorodiamidate linkages, have shown superior properties and several PMOs have entered clinical trials [43,44]. Carriers and targeting moieties such as cationic liposomes and cholesterol have been used to increase cellular uptake of anti-HBV ASOs successfully [42,45]. Polyethylenimine-based delivery of antisense oligonucleotides in a duck HBV (DHBV) model results in significant decrease in viremia accompanied by decrease in intrahepatic HBV DNA, RNA, Core and Surface proteins [46]. Hepatocyte targeting by attaching ASOs to ligands for receptor recognition can facilitate uptake by receptor-mediated endocytosis and effect significant inhibition of HBV replication [38,47].

## 3. Ribozymes Targeted to Hepatitis B Virus (HBV)

Ribozymes are catalytically active antisense RNA molecules. Their catalytic nature and ability to act independently of cellular pathways are the major attractions for use in gene therapy. In general, a potentially therapeutic ribozyme comprises an antisense sequence for specific RNA binding and a target cleaving enzymatic domain. Naturally occurring classes of ribozymes include Group I introns, Group II introns, Ribonuclease P (RNase P) and the hammerhead, hepatitis delta virus, hairpin and Neurospora Varkud satellite ribozymes. Group I introns, Group II introns and RNase P are larger and more structurally complex ribozymes. RNase P catalyzes the maturation of precursor tRNAs (pre-tRNAs) by cleaving the 5′ leader sequence, whereas Group I and II introns catalyze self-splicing to facilitate their own excision from pre-mRNAs, pre-tRNAs and pre-rRNAs. Hammerhead, hepatitis delta virus, hairpin and Neurospora Varkud satellite ribozymes are the smallest ribozymes and are involved in processing the products of rolling circle replication in viruses or satellite RNA genomes [20,48,49].

The antisense domains of ribozymes can be manipulated to bind sequences of interest. Among the larger ribozymes, RNase P is the most explored for use against viruses. RNase P ribozymes can be modified by linking the catalytic domain to an external guide sequence (EGS) that is complementary to the target. Binding of the EGS to mRNA forms a pre-tRNA-like structure [50,51]. A recent study by Xia *et al*. took advantage of the property of eukaryotic systems to naturally encode RNase P and designed an RNase P-free EGS that targeted the *pre-S1* and *surface* regions of the pgRNA. This resulted in recruitment of intracellular RNase P and significant suppression of viral gene expression with reduced viral DNA levels in cell culture and murine models of HBV replication [52].

Hairpin and hammerhead ribozymes have also been engineered to confer sequence-specific cleavage of HBV mRNA. Hairpin ribozymes targeting *core*, *S*, *pol* and *X* ORFs resulted in efficient target RNA cleavage and suppression of HBV replication in liver-derived cells [53,54]. Hammerhead ribozymes are the best characterized and most commonly used small ribozyme against HBV. Their structure comprises three base paired helices (Helix I, II, and III) separated by a conserved short linker sequences. Helix II is the catalytic domain, whereas Helix I and III hybridize to targets in a sequence-specific manner (Figure 2) [21]. Effective hammerhead ribozymes targeting the HBV packaging signal, polyadenylation signal, *S* and *X* ORFs have been mainly tested in cell culture [55,56,57,58,59] and in few mouse model studies [57,60]. Because hepatitis D virus (HDV) has the same tropism and requires HBV for replication, it has been used to facilitate targeted delivery of hammerhead ribozymes against the *X* and *core* sequences within the pgRNA. Reduction of HBV transcripts, HBeAg secretion and HBV genomes by ribozymes expressed from HDV was observed [61,62]. Hammerhead ribozymes lacking Helix II (minizymes) have been designed and shown to have catalytic activity. Minizymes can suppress hepatitis B surface antigen (HBsAg) expression by >80% and inhibit HBV DNA replication [63]. Although minizymes are much smaller and can be easily synthesized, they are mostly limited by their reduced activity compared to full-length ribozymes [64,65].

As compared to the anti-HBV DNA-based strategies discussed in this review, ribozymes generally have lower intracellular efficiency, which has delayed progress of anti-HBV ribozymes to clinical development. To improve activity, self-cleaving hammerhead ribozymes with defined sequences expressed as multimeric cassettes have been used and resulted in improved knockdown of HBV replication markers in cultured cells [66,67].

## 4. Manipulation of RNA Interference (RNAi) to Counter HBV Infection

RNAi is a highly conserved sequence-dependant gene-silencing pathway involved in regulating a wide range of cellular processes. The mechanism is initiated by the expression of primary microRNAs (pri-miRs) that include double-stranded RNA hairpin regions. Pri-miRs are processed in sequential nuclear and cytoplasmic cleavage steps that are catalyzed by cellular endonucleases. Pri-miRs are initially processed by the Drosha RNase III, which functions in partnership with a double-stranded RNA (dsRNA) binding partner, Di George Critical Region 8 (DGCR8) protein. This gives rise to precursor miRs (pre-miRs), which are transported to the cytoplasm for further cleavage by Dicer to yield mature miR duplexes of 21–23 bp. Mature miRs are then incorporated into an RNA inducing silencing complex (RISC) containing Argonaute 2 (Ago2). One strand is selected as a guide and the remaining strand (passenger/antiguide) is subsequently degraded or released from RISC. The guide strand hybridizes to target mRNA to promote degradation or translation inhibition (reviewed in [68,69,70], Figure 2).

Soon after the discovery of the RNAi pathway by Fire and Mello in 1998 [71], exploitation of this pathway for selective silencing of genes became the tool of choice in antiviral gene therapy. RNAi-based anti-HBV agents have progressed to a stage of testing in clinical trials [4,72,73]. Manipulation of RNAi for therapeutic purposes has been thoroughly investigated and is more effective than other nucleic-acid based antisense and ribozyme approaches. RNAi-mediated gene silencing is achieved using synthetic or expressed RNA sequences (RNAi activators) that resemble miR duplexes, pre-miR or pri-miR. Typically, synthetic activators are designed to resemble miR duplexes (short interfering RNAs/siRNAs), whereas expressed activators resemble pre-miR (short hairpin RNAs/shRNAs and pre-miR mimics) and pri-miR (pri-miR mimics). Advantages of using siRNAs include ease of production, dose control and chemical modification to increase stability, specificity and reduced immunostimulatory effects (reviewed in [74,75,76]). As they act in the cytoplasm and do not require nuclear delivery, efficient cellular delivery is easier to achieve than with DNA expression cassettes. siRNAs may be successfully delivered using less immunogenic non-viral vectors, and liposome delivery systems are the most commonly used [77,78,79].

Several siRNAs against HBV have entered clinical development. Recent pre-clinical studies with an RNAi based drug, ARC-520 developed by Arrowhead Research Corporation (Pasadena, CA, USA), showed promising results. ARC-520 comprises two cholesterol-conjugated siRNAs and a hepatocyte-targeted membrane-lytic-peptide (NAG-MLP). Injection of ARC-520 in a chronically infected chimpanzee decreased HBV DNA levels 36-fold, HBeAg by 10-fold and HBsAg by 80% after two injections [79,80]. Reports from Arrowhead Research Corporation showed that ARC-520 was safe and well tolerated in the Phase 1 clinical trial. The Phase 2a clinical trial in chronically infected patients showed significant HBsAg reduction and was well tolerated [81] (ClinicalTrials.gov ID: NCT02065336). Recently, Alnylam Pharmaceuticals (Cambridge, MA, USA) reported up to 4 log reduction in circulating HBV DNA in chronically infected chimpanzees injected with lipid nanoparticle formulations containing siRNA ESC-GalNAc-siRNA (Enhanced Stabilization Chemistry-*N*-Acetylgalactosamine-siRNA) [82]. Tekmira (TKM) Pharmaceuticals (Burnaby, British Columbia, Canada) recently reported a 1 log reduction in HBsAg in chronically infected humanized mice injected with TKM-HBV, a lipid nanoparticle containing three different siRNA [83]. Phase I clinical trials with TKM-HBV are now underway [84].

Despite the exciting developments in the use of siRNAs for HBV treatment, therapy requires repeated administration to prolong therapeutic effects. The prospects of using expressed shRNAs or pri-miR mimics for durable HBV inactivation have therefore been subject of extensive research, and significant progress has been made. Expressed shRNAs or pri-miR mimics have the advantage of sustained supply of RNAi activators and are hence more relevant for targeting chronic diseases such as HBV infection. However, several studies reported toxicities that were caused by saturation of the RNAi pathway following overexpression of shRNAs from RNA polymerase (Pol) III promoters [85,86,87]. Use of cassettes containing artificial pri-miRs may alleviate this problem as these templates may be expressed from Pol II promoters to afford better transcription control. The use of pri-miR mimics also enables design of polycistronic cassettes that produce multiple effectors with different target sites, a strategy that is useful to augment inhibition of gene expression and prevent viral escape [88,89,90]. However, the progress of expressed anti-HBV RNAi activators to clinic is hampered by lack of efficient, easily scalable and safe delivery vectors. Alternatives to the *in vivo* gene delivery of expressed anti-HBV sequences are currently being explored. Lentiviral, adenoviral and adeno-associated viral vectors (AAVs) have been evaluated with promising results [91,92,93]. Despite their limited packaging capacity, adeno-associated viral vectors are suitable for delivering smaller RNAi activators. AAVs are non-pathogenic and hence remain the most attractive vector for delivery of anti-HBV gene therapy. The development of AAVs that express three shRNAs against hepatitis C (TT-034) support the use of AAVs as anti-HBV gene therapy delivery vectors. This candidate drug has reached phase I/II clinical trials conducted by Benitec Biopharma (Balmain, Sydney, Australia) [94] (ClinicalTrials.gov ID: NCT01899092).

## 5. Genome and Epigenome Editing for HBV Gene Therapy

Genome editing is a technique that utilizes engineered DNA-binding nucleases for targeted gene replacement, addition, or inactivation. At the forefront of genome engineering is site-specific nuclease technology that has paved the way for genetic analysis and manipulation (reviewed in [95,96]). Engineered nucleases with modular DNA-binding domains or RNA-guided DNA targets may be employed to bind and digest specific DNA sequences to enable permanent disruption of target genes. Programmable nucleases cleave DNA sequences and induce double-strand breaks (DSBs) at the target sites. Subsequent repair mechanisms occur by pathways that entail homology-directed repair (HDR) or non-homologous end joining (NHEJ) (reviewed in [97,98]). HDR enables precise genetic modification by using donor template or exogenous oligonucleotide sequences that have flanking homologous regions [99]. Error-prone NHEJ has the potential to knock out genes by causing targeted small insertions or deletions (indels) [100]. Misrepair of HBV genes caused by this mechanism may result in inactivation of replication of the virus [24]. The three most commonly used designer nucleases that have been used for genetic modification are the ZFNs, TALENs and the RNA-guided CRISPR-Cas systems (reviewed in [101]). Here we describe each of these classes of engineered DNA-binding proteins and their potential for application to HBV gene therapy.

### 5.1. Zinc Finger Proteins and Their Derivatives

Zinc finger proteins (ZFP) are found in ~3% of human genes and were the first class of eukaryotic transcription factors to be used in genome editing (reviewed in [102]). ZFNs have been utilized widely for modifying the genomes of a variety of cells [103,104], including those of plants [105,106], fish [107], mice [108], ducks [109] and humans [110,111,112]. ZFP domains comprise three to six zinc finger modules that each consists of 30 amino acids. These modules contain conserved Cys_2_His_2_ zinc finger motifs, which recognize specific base triplets in the major groove of DNA (reviewed in [113]). By engineering tandem modules, longer nucleotide sequences of 9 to 18 base pairs may be targeted with high specificity [114]. ZFPs may be modified to confer nuclease or transcriptional repressor functions. Typically, modular ZFPs are engineered to form ZFNs by fusing the DNA-binding domain to a single-strand DNA nuclease, such as that from the *Fok*I type IIS restriction enzyme. The endonuclease is directed to create a nick in a strand of the DNA at the sites targeted by the ZFP [96]. ZFNs are designed to bind to opposite DNA strands and the DNA-binding domains are typically separated by a spacer of five to seven nucleotides. Cleavage of duplex DNA thus occurs when two ZFN monomers bind to adjacent target sites on the sense and antisense strands (Figure 3) (reviewed in [113]). A ZFN pair which specifically recognizes 18 to 36 base pairs may thus be engineered to be unique and specific for the gene of interest. Although off-target effects may be minimal [24,103], a limitation of ZFNs is that DNA binding efficiency of the modules is influenced by the neighboring zinc finger domains. Also, modules that bind to each of the 64 possible nucleotide triplets of DNA have not yet been described, which means that currently it is not possible to generate a ZFN that recognizes any DNA sequence. Empirical evaluation is crucial to enable selection of optimally acting ZFNs [97]. TALENs and CRISPR-Cas derivatives are not encumbered by these problems and these endonucleases have therefore gained favor for therapeutic gene editing.

### 5.2. Transcription Activator-Like Effectors and Their Derivatives

The general structure of TALENs is analogous to that of ZFNs in that a *Fok*I cleavage domain is coupled to the carboxyl terminus of the DNA-binding modules [96]. The sequence-specific properties of TALENs consist of derivatives of the transcription activator-like effector (TALE) proteins, which are secreted by the *Xanthomonas* or *Ralstonia* species of phytopathogenic bacteria [115]. Each TALE has an N-terminal nuclear localization signal, a C-terminal effector domain, and a DNA-binding domain that comprises tandem-repeats. Each module of the repetitive region is made up of a sequence of 33 to 35 amino acids and recognizes one nucleotide of a DNA base pair. Specific interaction with each nucleotide occurs through binding of repeat variable diresidues (RVDs), which are situated at amino acids 12 and 13 of the TALENs’ monomers. Deciphering the code of the RVDs has enabled engineering of sequence-specific DNA binding proteins. Commonly used RVDs that recognize adenine, guanine, cytosine and thymine are Asn-Ile, Asn-Asn, His-Asp and Asn-Gly, respectively. An important advantage that TALE derivatives have over ZFPs is that the binding of the individual modules to their cognates is not influenced by neighboring monomers (Figure 3) (reviewed in [116,117]).

TALENs, like ZFNs, generate DSBs at an intended target site and can be applied in a similar manner to knock out genes or knock in mutations [96]. A requirement of TALEN design is that a thymine residue should be present at the 5′ end of the target sequence [118]. Each monomer of a TALEN typically is encoded by approximately 3 kb of DNA, which is larger than the sequences required to encode ZFN monomers. This large size of the expression cassettes poses challenges for packaging and delivery using viral vectors of limited transgene capacity [119]. If efficient delivery to hepatocytes with gene transfer vectors can be linked with precise targeting of the HBV cccDNA, the use of TALENs is potentially curative of HBV infection. Targeted HBV transcriptional repression, without cleavage of the DNA, can potentially be achieved with repressor TALEs (rTALEs). rTALEs may be generated from TALEs by fusion of a transcriptional repressor domain, such as the Krüppel-associated box (KRAB). Naturally occurring KRAB domains are typically associated with ZFPs and comprise ~75 amino acid residues [120]. They mainly function through protein-protein interactions and recruitment of various heterochromatin-inducing factors by KRAB-associated protein 1 (KAP1). Epigenome-modifying proteins, such as histone-modifying protein 1 (HP1), form key mediators of a strong transcriptional inhibitory effect [120,121]. Repressor TALE technology is still in its infancy and molecular or preclinical research has been limited. However, the potential for a protein effector to target HBV DNA sequences with high specificity, allowing for inhibition of viral transcription machinery without causing mutations in the genome, presents advantages for effective antiviral gene therapy.

### 5.3. CRISPR-Cas

The most recent genome editing technology is derived from the RNA-guided CRISPR-Cas system, which is a form of prokaryotic acquired immunity that defends against invading phages and plasmids. Engineered CRISPR-Cas systems are distinct from ZFNs and TALENs in that RNA-DNA base pairing is responsible for conferring specificity on the Cas nuclease. Diverse CRISPR-Cas systems have been identified, which are distinguished by their *cas* genes and the structure and functioning of their Cas proteins (reviewed in [122,123,124,125]). The CRISPR locus contains a direct repeat array of similar sequences of 24 to 48 nucleotides that are interspersed with variable spacer sequences of 26 to 72 bases. The system also comprises regions coding for transactivating CRISPR RNA (tracrRNA) and Cas proteins [123]. An essential feature of Type II CRISPR-Cas systems is that foreign DNA protospacers are incorporated into bacterial genomic sequences and transcribed into CRISPR RNA (crRNA) [122]. During inactivation of invading DNA the crRNA anneals to tracrRNA to form short chimeric RNA molecules. These single guide transcripts (sgRNAs) then direct the Cas endonuclease to effect sequence-specific cleavage of pathogenic DNA that is complementary to the protospacer sequences (Figure 3) [122,123]. A significant advance in employing CRISPR-Cas technology to gene editing has been the combining of crRNA and tracrRNA components into sgRNA. By using sgRNA, artificial CRISPR-Cas systems that comprise two instead of three components may be used, which simplifies the engineering of artificial nucleases. The Cas9 protein requires an upstream protospacer adjacent motif (PAM) for target cleavage [125]. In the case of the commonly used Cas9 from *Streptococcus pyogenes,* the canonical sequence is 5′-NGG-3′ in the DNA strand that is non-complementary to the crRNA. The delivery of sgRNAs in combination with the simplicity of engineering CRISPR-Cas effector molecules has made this versatile nuclease technology a popular choice for therapeutic gene editing.

### 5.4. Application of Genome Editing to HBV Therapy

To enable application of genome editing to treatment of HBV infection, efficient hepatotropic delivery of gene editors that are capable of eradicating the long-lived episomal cccDNA is important. The group of Zimmerman *et al.* was the first to advance a gene editing approach to countering HBV replication [109]. The researchers used DHBV as a model and designed six different ZFPs to target the DHBV enhancer sequences; A region in the cccDNA, which controls transcription of the *core* and *surface* sequences. Viral transcription was measured following co-transfection of a plasmid encoding the ZFP and DHBV replication-competent DNA. A marked decrease in viral pgRNA and total viral RNA was recorded for those cells expressing the ZFPs. Moreover, ZFPs significantly reduced viral Core and Surface protein production without any noticeable cytotoxicity. Although inhibition was impressive, a lasting effect was unlikely as the ZFPs did not cause targeted DNA mutation or introduce durable epigenetic changes. Subsequent work of Cradick and colleagues demonstrated the utility of ZFNs for targeted cleavage of HBV episomal DNA sequences [24]. The team engineered nine pairs of HBV-specific three-finger ZFNs and co-transfected hepatoma cells with each ZFN pair plus an HBV genome target plasmid. Targeted cleavage of the viral sequences was observed with concomitant inhibition of markers of viral replication. Although encouraging, the study did not demonstrate that cccDNA was modified by the engineered ZFNs.

As the *X* gene is thought to play a central role in hepatocarcinogenesis (reviewed in [126]), Zhao *et al.* used ZFP-based technology to inhibit expression of integrated sequences of this gene [22]. An artificial transcription factor (ATF) was designed to target an 18 bp sequence in the enhancer 1 region, which is upstream of the integrated *X* promoter. The ATF comprised a DNA-binding domain of a ZFP that was linked to a KRAB repressor domain. Results from a luciferase reporter assay showed *X* repression, but this approach has not been developed to evaluation in a preclinical setting. A recent study by Weber *et al.* also applied ZFNs to develop anti-HBV drug therapy [127]. The aim was to prevent viral reactivation by targeting three HBV protein-coding sequences in HepAD38 cells. Self-complementary adeno-associated viral vectors (scAAVs) containing sequences encoding the ZFNs were used to deliver the engineered gene editors. Site-specific mutagenesis was confirmed and low cytotoxicity was observed for two of the three ZFNs. The most striking observation of the study was that inhibition of viral replication and particle production over a period of 14 days could be achieved after a single treatment with the scAAVs encoding a ZFN targeted to the viral *pol* ORF.

Recently, Bloom *et al.* were the first to validate the use of TALENs to disable HBV replication in cultured cells and *in vivo* [26]. Four HBV-specific TALENs were generated that targeted conserved sequences in the *surface* (S TALEN), *core* (C TALEN) and *pol* (P1 and P2 TALENs) ORFs. The TALENs targeting *surface* and *core* sequences were most effective in cultured cells and *in vivo*. A Hirt’s DNA extraction coupled to treatment of the extracts with ATP-dependent DNase, which degrades DNA with free 5′ or 3′ ends, was used to isolate putative viral cccDNA. Analysis employing a T7 endonuclease I (T7EI) assay verified targeted mutation in cultured cells. Hydrodynamic injection of mice was used to simulate HBV replication *in vivo*. Substantial decreases in viral replication markers were observed: The S TALEN reduced HBsAg by more than 90% and circulating viral particle equivalents were diminished by approximately 70% by the S and C TALENs. T7E1 assays and deep sequencing confirmed the targeted disruption. A more recent study by Chen *et al.* confirmed the successful targeting and inactivation of HBV genomic sequences by TALENs [128]. The researchers showed significant knockdown in markers of viral replication. Interestingly when used in combination with interferon-α, a licensed treatment of HBV infection, synergistic antiviral effects were observed. Although promising, a limitation of using mice to simulate HBV replication *in vivo* is that these animals do not produce the viral cccDNA replication intermediate.

A number of key studies employing CRISPR-Cas recently demonstrated the utility of RNA-guided cleavage of HBV DNA [28,129,130,131]. Lin *et al.* designed eight HBV-targeting sgRNAs [129]. A significant decrease in production of viral proteins was observed. Moreover co-transfection with more than one sgRNA-encoding sequence augmented antiviral efficacy. This effect was corroborated by an increase in indels at the targeted sites. Although the study reported on cleavage of HBV DNA sequences using combinations of sgRNA and Cas9 protein, efficacy against cccDNA in human cells was not evaluated. Seeger and Sohn investigated targeted disruption of HBV cccDNA and confirmed efficient cleavage of viral sequences with all five of their sgRNA constructs [130]. On average, an eight-fold inhibition of HBcAg expression was recorded in HBV-infected HepG2/NTCP cells. To deliver the engineered CRISPR-Cas sequences, recombinant lentiviral vectors were used to transduce the cells. Targeted mutations included single-nucleotide indels and large deletions up to 2.3 kb. The potential of CRISPR-Cas to effect such large mutations suggests that targeting and excision of host-integrated HBV genomes may be feasible. A subsequent study by Kennedy *et al.* also showed effective inhibition of HBV replication following delivery of Cas9 and sgRNA sequences with recombinant lentiviral vectors [28]. The research team of Dong *et al.* confirmed efficacy of sgRNA-Cas9 against HBV. Interestingly they also demonstrated disruption of artificial cccDNA in a murine hydrodynamic model [131] that was based on use of engineered recombinant cccDNA precursor plasmid (rcccDNA) [132].

Advances in employing designer endonucleases to disable HBV replication are encouraging. However, a fundamental concern for using endonucleases to mediate gene disruption is potential for off-target effects [127]. Resultant mutations may have detrimental consequences, and careful consideration needs to be given to the design and characterization of engineered nucleases. Artificial HBV-targeting nucleases also may cleave HBV target sequences that have been integrated into the host genome. This may pose a risk for chromosomal translocation, and use of rTALEs, which do not have nuclease function, may limit this effect.

## 6. Conclusions and Future Perspectives

Development of an affordable therapy that will result in complete clearance of HBV in infected individuals still remains a challenge. Application of gene therapy is one of the approaches that have the potential to achieve this goal. Because of the inconsistent efficiencies observed in the majority of studies using ribozymes to counter HBV infection, this class of viral gene inhibitors has not progressed to clinical trial for evaluating efficacy of treatment of the virus. However, several ASO and siRNA formulations showed promising outcomes in different clinical trials. HBV gene editing by designer endonucleases, such as TALENs and CRISPR-Cas, has attracted considerable interest during the last few years. Although there is not yet enough information to confirm the efficacy and safety of these nucleases in models that mimic human disease more closely (e.g., non-human primates), this technology promises to overcome the challenge of HBV cccDNA persistence and the resultant relapse after treatment. Application of gene editing to treatment of HBV infection thus offers a realistic prospect of establishing curative therapy for chronic HBV infection.

Despite these exciting developments, the use of gene silencing and editing technologies for the treatment of chronic infections faces a number of hurdles. These relate to considerations of stability of the effectors, toxicity, target specificity, prevention of viral escape and suitability for delivery *in vivo*. Immense efforts to address these challenges are leading to innovative solutions with concomitant progress in developing gene therapy for chronic HBV infection. Combinatorial gene silencing, for example, using sequences targeting multiple regions of the viral genome, holds promise for augmented silencing and reducing the probability of viral escape. This is supported by promising data from both ARC-520 and TKM-HBV formulations, which contain three virus-targeting siRNAs. Reports by Arrowhead Research Corporation (Pasadena, CA, USA) show that combination of ARC-520 with entecavir in mice results in a synergistic effect [133]. Combination of siRNA and ASOs targeting the α-2,6-sialyltransferase (*ST6Gal I*) gene in cell culture results in synergistic and additive gene silencing [134]. The combination of different anti-HBV strategies may therefore also contribute significantly to the elimination of HBV in infected individuals.

The ability to fine-tune nucleic acid-based therapeutics for improved specificity and activity makes gene therapy an exciting new approach to combating viral infections. Rapid advances in biotechnology and expanding knowledge about the HBV life cycle, viral diversity and interaction with the host factors will continue to facilitate discovery of novel therapeutics. Lead anti-HBV nucleic acid-based candidate drugs will continue to enter clinical development. Integrating currently available information, and perhaps use of gene therapy in combination with currently licensed therapies, may result in superior treatment of chronic HBV infection. Directing focus to engineering efficient, safe and cost effective delivery systems will be crucial to facilitating progress of anti-HBV gene therapy in the clinic.

## References

[1] Franco E., Bagnato B., Marino M.G., Meleleo C., Serino L., Zaratti L. (2012). Hepatitis B: Epidemiology and prevention in developing countries. World J. Hepatol..

[2] Montuclard C., Hamza S., Rollot F., Evrard P., Faivre J., Hillon P., Martino V.D., Minello A. (2015). Causes of death in people with chronic HBV infection: A population-based cohort study. J. Hepatol..

[3] Arbuthnot P., Carmona S., Ely A. (2005). Exploiting the RNA interference pathway to counter hepatitis B virus replication. Liver Int..

[4] Ivacik D., Ely A., Arbuthnot P. (2011). Countering hepatitis B virus infection using RNAi: How far are we from the clinic?. Rev. Med. Virol..

[5] Karayiannis P. (2003). Hepatitis B virus: Old, new and future approaches to antiviral treatment. J. Antimicrob. Chemother..

[6] Xu B., Lin L., Xu G., Zhuang Y., Guo Q., Liu Y., Wang H., Zhou X., Wu S., Bao S. (2015). Long-term lamivudine treatment achieves regression of advanced liver fibrosis/cirrhosis in patients with chronic hepatitis B. J. Gastroenterol. Hepatol..

[7] Gebbing M., Bergmann T., Schulz E., Ehrhardt A. (2015). Gene therapeutic approaches to inhibit hepatitis B virus replication. World J. Hepatol..

[8] Schulze A., Gripon P., Urban S. (2007). Hepatitis B virus infection initiates with a large surface protein-dependent binding to heparan sulfate proteoglycans. Hepatology.

[9] Yan H., Zhong G., Xu G., He W., Jing Z., Gao Z., Huang Y., Qi Y., Peng B., Wang H. (2012). Sodium taurocholate cotransporting polypeptide is a functional receptor for human hepatitis B and D virus. eLife.

[10] Katen S.P., Tan Z., Chirapu S.R., Finn M.G., Zlotnick A. (2013). Assembly-directed antivirals differentially bind quasiequivalent pockets to modify hepatitis B virus capsid tertiary and quaternary structure. Structure.

[11] Feld J.J., Colledge D., Sozzi V., Edwards R., Littlejohn M., Locarnini S.A. (2007). The phenylpropenamide derivative AT-130 blocks HBV replication at the level of viral RNA packaging. Antivir. Res..

[12] Delaney W.E.T., Edwards R., Colledge D., Shaw T., Furman P., Painter G., Locarnini S. (2002). Phenylpropenamide derivatives AT-61 and AT-130 inhibit replication of wild-type and lamivudine-resistant strains of hepatitis B virus *in vitro*. Antimicrob. Agents Chemother..

[13] Yang X.Y., Xu X.Q., Guan H., Wang L.L., Wu Q., Zhao G.M., Li S. (2014). A new series of HAPs as anti-HBV agents targeting at capsid assembly. Bioorg. Med. Chem. Lett..

[14] Yan H., Peng B., He W., Zhong G., Qi Y., Ren B., Gao Z., Jing Z., Song M., Xu G. (2013). Molecular determinants of hepatitis B and D virus entry restriction in mouse sodium taurocholate cotransporting polypeptide. J. Virol..

[15] Zhong G., Yan H., Wang H., He W., Jing Z., Qi Y., Fu L., Gao Z., Huang Y., Xu G. (2013). Sodium taurocholate cotransporting polypeptide mediates woolly monkey hepatitis B virus infection of tupaia hepatocytes. J. Virol..

[16] Watashi K., Urban S., Li W., Wakita T. (2014). NTCP and beyond: Opening the door to unveil hepatitis B virus entry. Int. J. Mol. Sci..

[17] Ding X., Yang J., Wang S. (2011). Antisense oligonucleotides targeting abhydrolase domain containing 2 block human hepatitis B virus propagation. Oligonucleotides.

[18] Ketting R.F. (2011). MicroRNA biogenesis and function: An overview. Adv. Exp. Med. Biol..

[19] Hasselblatt P., Hockenjos B., Thoma C., Blum H.E., Offensperger W.B. (2005). Translation of stable hepadnaviral mRNA cleavage fragments induced by the action of phosphorothioate-modified antisense oligodeoxynucleotides. Nucleic Acids Res..

[20] Khan A.U. (2006). Ribozyme: A clinical tool. Clin. Chim. Acta.

[21] Doherty E.A., Doudna J.A. (2001). Ribozyme structures and mechanisms. Annu. Rev. Biophys. Biomol. Struct..

[22] Zhao X., Zhao Z., Guo J., Huang P., Zhu X., Zhou X., Yang Z., Zhao L., Xu L., Xu J. (2013). Creation of a six-fingered artificial transcription factor that represses the hepatitis B virus *HBx* gene integrated into a human hepatocellular carcinoma cell line. J. Biomol. Screen..

[23] Li G., Jiang G., Lu J., Chen S., Cui L., Jiao J., Wang Y. (2014). Inhibition of hepatitis B virus cccDNA by siRNA in transgenic mice. Cell Biochem. Biophys..

[24] Cradick T.J., Keck K., Bradshaw S., Jamieson A.C., McCaffrey A.P. (2010). Zinc-finger nucleases as a novel therapeutic strategy for targeting hepatitis B virus DNAs. Mol. Ther..

[25] Wu Y., Gao T., Wang X., Hu Y., Hu X., Hu Z., Pang J., Li Z., Xue J., Feng M. (2014). TALE nickase mediates high efficient targeted transgene integration at the human multi-copy ribosomal DNA locus. Biochem. Biophys. Res. Commun..

[26] Bloom K., Ely A., Mussolino C., Cathomen T., Arbuthnot P. (2013). Inactivation of hepatitis B virus replication in cultured cells and *in vivo* with engineered transcription activator-like effector nucleases. Mol. Ther..

[27] Zhu W., Lei R., Le Duff Y., Li J., Guo F., Wainberg M.A., Liang C. (2015). The CRISPR-cas9 system inactivates latent HIV-1 proviral DNA. Retrovirology.

[28] Kennedy E.M., Bassit L.C., Mueller H., Kornepati A.V., Bogerd H.P., Nie T., Chatterjee P., Javanbakht H., Schinazi R.F., Cullen B.R. (2015). Suppression of hepatitis B virus DNA accumulation in chronically infected cells using a bacterial CRISPR-cas RNA-guided DNA endonuclease. Virology.

[29] Zhen S., Hua L., Liu Y.H., Gao L.C., Fu J., Wan D.Y., Dong L.H., Song H.F., Gao X. (2015). Harnessing the clustered regularly interspaced short palindromic repeat (CRISPR)/CRISPR-associated Cas9 system to disrupt the hepatitis B virus. Gene Ther..

[30] Van Hauwermeiren F., Vandenbroucke R.E., Grine L., Puimege L., van Wonterghem E., Zhang H., Libert C. (2014). Antisense oligonucleotides against TNFR1 prevent toxicity of TNF/IFNγ treatment in mouse tumor models. Int. J. Cancer.

[31] Goodarzi G., Gross S.C., Tewari A., Watabe K. (1990). Antisense oligodeoxyribonucleotides inhibit the expression of the gene for hepatitis B virus surface antigen. J. Gen. Virol..

[32] Wu G.Y., Wu C.H. (1992). Specific inhibition of hepatitis B viral gene expression in vitro by targeted antisense oligonucleotides. J. Biol. Chem..

[33] Offensperger W.B., Offensperger S., Walter E., Teubner K., Igloi G., Blum H.E., Gerok W. (1993). *In vivo* inhibition of duck hepatitis B virus replication and gene expression by phosphorothioate modified antisense oligodeoxynucleotides. EMBO J..

[34] Yao Z., Zhou Y., Feng X., Chen C., Guo J. (1996). *In vivo* inhibition of hepatitis B viral gene expression by antisense phosphorothioate oligodeoxynucleotides in athymic nude mice. J. Viral Hepat..

[35] Yao Z.Q., Zhou Y.X., Guo J., Feng Z.H., Feng X.M., Chen C.X., Jiao J.Z., Wang S.Q. (1996). Inhibition of hepatitis B virus *in vitro* by antisense oligonucleotides. Acta Virol..

[36] Deng Y.B., Nong L.G., Huang W., Pang G.G., Wang Y.F. (2009). Inhibition of hepatitis B virus (HBV) replication using antisense LNA targeting to both *S* and *C* genes in HBV. Zhonghua Gan Zang Bing Za Zhi.

[37] Korba B.E., Gerin J.L. (1995). Antisense oligonucleotides are effective inhibitors of hepatitis B virus replication *in vitro*. Antivir. Res..

[38] Nakazono K., Ito Y., Wu C.H., Wu G.Y. (1996). Inhibition of hepatitis B virus replication by targeted pretreatment of complexed antisense DNA *in vitro*. Hepatology.

[39] De Smet M.D., Meenken C.J., van den Horn G.J. (1999). Fomivirsen—A phosphorothioate oligonucleotide for the treatment of CMV retinitis. Ocul. Immunol. Inflamm..

[40] Jensen K.D., Kopeckova P., Kopecek J. (2002). Antisense oligonucleotides delivered to the lysosome escape and actively inhibit the hepatitis B virus. Bioconjug. Chem..

[41] Prakash T.P., Siwkowski A., Allerson C.R., Migawa M.T., Lee S., Gaus H.J., Black C., Seth P.P., Swayze E.E., Bhat B. (2010). Antisense oligonucleotides containing conformationally constrained 2′,4′-(*N*-methoxy)aminomethylene and 2′,4′-aminooxymethylene and 2′-*O*,4′-*C*-aminomethylene bridged nucleoside analogues show improved potency in animal models. J. Med. Chem..

[42] Deng Y., Nong L., Wei Y. (2013). Antiviral effects of dual-target antisense LNA by cationic liposomes in transgenic mice. Sheng Wu Yi Xue Gong Cheng Xue Za Zhi.

[43] Sun X., Marque L.O., Cordner Z., Pruitt J.L., Bhat M., Li P.P., Kannan G., Ladenheim E.E., Moran T.H., Margolis R.L. (2014). Phosphorodiamidate morpholino oligomers suppress mutant huntingtin expression and attenuate neurotoxicity. Hum. Mol. Genet..

[44] Cirak S., Arechavala-Gomeza V., Guglieri M., Feng L., Torelli S., Anthony K., Abbs S., Garralda M.E., Bourke J., Wells D.J. (2011). Exon skipping and dystrophin restoration in patients with duchenne muscular dystrophy after systemic phosphorodiamidate morpholino oligomer treatment: An open-label, phase 2, dose-escalation study. Lancet.

[45] Zhang Y., Qi X.R., Gao Y., Wei L. (2006). Transfection and anti-HBV effect mediated by the hepatocytes-targeting cationic liposomes co-modified with β-sitosterol-β-d-glucoside and Brij 35. Yao Xue Xue Bao.

[46] Robaczewska M., Guerret S., Remy J.S., Chemin I., Offensperger W.B., Chevallier M., Behr J.P., Podhajska A.J., Blum H.E., Trepo C. (2001). Inhibition of hepadnaviral replication by polyethylenimine-based intravenous delivery of antisense phosphodiester oligodeoxynucleotides to the liver. Gene Ther..

[47] Wu G.Y., Walton C.M., Wu C.H. (2001). Targeted polynucleotides for inhibition of hepatitis B and C viruses. Croat. Med. J..

[48] Wang C.X., Lu Y.Q., Qi P., Chen L.H., Han J.X. (2010). Efficient inhibition of hepatitis B virus replication by hepatitis delta virus ribozymes delivered by targeting retrovirus. Virol. J..

[49] Fedor M.J. (2000). Structure and function of the hairpin ribozyme. J. Mol. Biol..

[50] Yang Z., Vu G.P., Qian H., Chen Y.C., Wang Y., Reeves M., Zen K., Liu F. (2014). Engineered RNase P ribozymes effectively inhibit human cytomegalovirus gene expression and replication. Viruses.

[51] Bagheri S., Kashani-Sabet M. (2004). Ribozymes in the age of molecular therapeutics. Curr. Mol. Med..

[52] Xia C., Chen Y.C., Gong H., Zeng W., Vu G.P., Trang P., Lu S., Wu J., Liu F. (2013). Inhibition of hepatitis B virus gene expression and replication by ribonuclease P. Mol. Ther..

[53] Song Y.H., Lin J.S., Liu N.Z., Kong X.J., Xie N., Wang N.X., Jin Y.X., Liang K.H. (2002). Anti-HBV hairpin ribozyme-mediated cleavage of target RNA *in vitro*. World J. Gastroenterol..

[54] Welch P.J., Tritz R., Yei S., Barber J., Yu M. (1997). Intracellular application of hairpin ribozyme genes against hepatitis B virus. Gene Ther..

[55] Passman M., Weinberg M., Kew M., Arbuthnot P. (2000). *In situ* demonstration of inhibitory effects of hammerhead ribozymes that are targeted to the hepatitis Bx sequence in cultured cells. Biochem. Biophys. Res. Commun..

[56] Nash K.L., Alexander G.J., Lever A.M. (2005). Inhibition of hepatitis B virus by lentiviral vector delivered antisense RNA and hammerhead ribozymes. J. Viral Hepat..

[57] Morrissey D.V., Lee P.A., Johnson D.A., Overly S.L., McSwiggen J.A., Beigelman L., Mokler V.R., Maloney L., Vargeese C., Bowman K. (2002). Characterization of nuclease-resistant ribozymes directed against hepatitis B virus RNA. J. Viral Hepat..

[58] Feng Y., Kong Y.Y., Wang Y., Qi G.R. (2001). Inhibition of hepatitis B virus by hammerhead ribozyme targeted to the poly(a) signal sequence in cultured cells. Biol. Chem..

[59] Feng Y., Kong Y.Y., Wang Y., Qi G.R. (2001). Intracellular inhibition of the replication of hepatitis B virus by hammerhead ribozymes. J. Gastroenterol. Hepatol..

[60] Pan W.H., Xin P., Morrey J.D., Clawson G.A. (2004). A self-processing ribozyme cassette: Utility against human papillomavirus 11 E6/E7 mRNA and hepatitis B virus. Mol. Ther..

[61] Li X., Kuang E., Dai W., Zhou B., Yang F. (2005). Efficient inhibition of hepatitis B virus replication by hammerhead ribozymes delivered by hepatitis delta virus. Virus Res..

[62] Wen S.J., Xiang K.J., Huang Z.H., Zhou R., Qi X.Z. (2000). Construction of HBV-specific ribozyme and its recombinant with HDV and their cleavage activity *in vitro*. World J. Gastroenterol..

[63] Tan T.M., Zhou L., Houssais S., Seet B.L., Jaenicke S., Peter F., Lim S.G. (2002). Intracellular inhibition of hepatitis B virus *S* gene expression by chimeric DNA-RNA phosphorothioate minimized ribozyme. Antisense Nucleic Acid Drug Dev..

[64] Sioud M., Opstad A., Hendry P., Lockett T.J., Jennings P.A., McCall M.J. (1997). A minimised hammerhead ribozyme with activity against interleukin-2 in human cells. Biochem. Biophys. Res. Commun..

[65] McCall M.J., Hendry P., Jennings P.A. (1992). Minimal sequence requirements for ribozyme activity. Proc. Natl. Acad. Sci. USA.

[66] Weinberg M.S., Ely A., Passman M., Mufamadi S.M., Arbuthnot P. (2007). Effective anti-hepatitis B virus hammerhead ribozymes derived from multimeric precursors. Oligonucleotides.

[67] Ruiz J., Wu C.H., Ito Y., Wu G.Y. (1997). Design and preparation of a multimeric self-cleaving hammerhead ribozyme. BioTechniques.

[68] Bartel D.P. (2009). MicroRNAs: Target recognition and regulatory functions. Cell.

[69] Hammond S.M. (2005). Dicing and slicing: The core machinery of the rna interference pathway. FEBS Lett..

[70] Shabalina S.A., Koonin E.V. (2008). Origins and evolution of eukaryotic RNA interference. Trends Ecol. Evol..

[71] Fire A., Xu S., Montgomery M.K., Kostas S.A., Driver S.E., Mello C.C. (1998). Potent and specific genetic interference by double-stranded RNA in *Caenorhabditis elegans*. Nature.

[72] Angaji S.A., Hedayati S.S., Poor R.H., Madani S., Poor S.S., Panahi S. (2010). Application of RNA interference in treating human diseases. J. Genet..

[73] Haussecker D., Kay M.A. (2015). RNA interference. Drugging RNAi. Science.

[74] Chen X., Qian Y., Yan F., Tu J., Yang X., Xing Y., Chen Z. (2013). 5′-Triphosphate-siRNA activates RIG-I-dependent type I interferon production and enhances inhibition of hepatitis B virus replication in HepG2.2.15 cells. Eur. J. Pharmacol..

[75] Valenzuela R.A., Suter S.R., Ball-Jones A.A., Ibarra-Soza J.M., Zheng Y., Beal P.A. (2015). Base modification strategies to modulate immune stimulation by an siRNA. ChemBioChem.

[76] Xu L., Wang X., He H., Zhou J., Li X., Ma H., Li Z., Zeng Y., Shao R., Cen S. (2015). Structure-based design of novel chemical modification of the 3′-overhang for optimization of short interfering RNA performance. Biochemistry.

[77] Huang W., Li X., Yi M., Zhu S., Chen W. (2014). Targeted delivery of siRNA against hepatitis B virus by preS1 peptide molecular ligand. Hepatol. Res..

[78] Kong W.H., Bae K.H., Jo S.D., Kim J.S., Park T.G. (2012). Cationic lipid-coated gold nanoparticles as efficient and non-cytotoxic intracellular siRNA delivery vehicles. Pharm. Res..

[79] Wooddell C.I., Rozema D.B., Hossbach M., John M., Hamilton H.L., Chu Q., Hegge J.O., Klein J.J., Wakefield D.H., Oropeza C.E. (2013). Hepatocyte-targeted RNAi therapeutics for the treatment of chronic hepatitis B virus infection. Mol. Ther..

[80] Sebestyen M.G., Wong S.C., Trubetskoy V., Lewis D.L., Wooddell C.I. (2015). Targeted *in vivo* delivery of siRNA and an endosome-releasing agent to hepatocytes. Methods Mol. Biol..

[81] Arrowhead Presents Data on ARC-520 and ARC-AAT at AASLD the Liver Meeting^®^ 2014. http://ir.arrowheadresearch.com/releasedetail.cfm?releaseid=881791.

[82] Alnylam Announces New RNAi Therapeutic Program for the Treatment of Hepatitis B Virus (HBV) Infection and Reports an Up to 2.3 Log10 Reduction of HBV Surface Antigen (HBsAg) in Chronically Infected Chimpanzees. http://investors.alnylam.com/releasedetail.cfm?ReleaseID=847055.

[83] Tekmira Presents Results of Preclinical Studies with Hepatitis B Therapeutic. http://www.sec.gov/Archives/edgar/data/1447028/000117184314004780/newsrelease.htm.

[84] Tekmira Initiates Phase I Clinical Trial of TKM-HBV. http://investor.tekmirapharm.com/releasedetail.cfm?ReleaseID=892233.

[85] Evaluation of TT-034: Safe and Durable Hepatic Expression of Anti-HCV ShRNA in a Non-Human Primate Model. http://www.benitec.com/documents/1311_tt_presn_hcv_aasld.pdf.

[86] Grimm D., Streetz K.L., Jopling C.L., Storm T.A., Pandey K., Davis C.R., Marion P., Salazar F., Kay M.A. (2006). Fatality in mice due to oversaturation of cellular microRNA/short hairpin RNA pathways. Nature.

[87] Sun C.P., Wu T.H., Chen C.C., Wu P.Y., Shih Y.M., Tsuneyama K., Tao M.H. (2013). Studies of efficacy and liver toxicity related to adeno-associated virus-mediated RNA interference. Hum. Gene Ther..

[88] Grimm D., Wang L., Lee J.S., Schurmann N., Gu S., Borner K., Storm T.A., Kay M.A. (2010). Argonaute proteins are key determinants of RNAi efficacy, toxicity, and persistence in the adult mouse liver. J. Clin. Investig..

[89] McBride J.L., Boudreau R.L., Harper S.Q., Staber P.D., Monteys A.M., Martins I., Gilmore B.L., Burstein H., Peluso R.W., Polisky B. (2008). Artificial miRNAs mitigate shRNA-mediated toxicity in the brain: Implications for the therapeutic development of RNAi. Proc. Natl. Acad. Sci. USA.

[90] Ely A., Naidoo T., Arbuthnot P. (2009). Efficient silencing of gene expression with modular trimeric Pol II expression cassettes comprising microRNA shuttles. Nucleic Acids Res..

[91] Ely A., Naidoo T., Mufamadi S., Crowther C., Arbuthnot P. (2008). Expressed anti-HBV primary microRNA shuttles inhibit viral replication efficiently *in vitro* and *in vivo*. Mol. Ther..

[92] Ivacik D., Ely A., Ferry N., Arbuthnot P. (2015). Sustained inhibition of hepatitis B virus replication *in vivo* using RNAi-activating lentiviruses. Gene Ther..

[93] Mowa M.B., Crowther C., Ely A., Arbuthnot P. (2014). Inhibition of hepatitis B virus replication by helper dependent adenoviral vectors expressing artificial anti-HBV pri-miRs from a liver-specific promoter. Biomed. Res. Int..

[94] Giering J.C., Grimm D., Storm T.A., Kay M.A. (2008). Expression of shRNA from a tissue-specific Pol II promoter is an effective and safe RNAi therapeutic. Mol. Ther..

[95] Gaj T., Gersbach C.A., Barbas C.F. (2013). ZFN, TALEN, and CRISPR-cas-based methods for genome engineering. Trends Biotechnol..

[96] Kim H., Kim J.S. (2014). A guide to genome engineering with programmable nucleases. Nat. Rev. Genet..

[97] Urnov F.D., Rebar E.J., Holmes M.C., Zhang H.S., Gregory P.D. (2010). Genome editing with engineered zinc finger nucleases. Nat. Rev. Genet..

[98] Gupta R.M., Musunuru K. (2014). Expanding the genetic editing tool kit: ZFNs, TALENs, and CRISPR-Cas9. J. Clin. Investig..

[99] Chu V.T., Weber T., Wefers B., Wurst W., Sander S., Rajewsky K., Kuhn R. (2015). Increasing the efficiency of homology-directed repair for CRISPR-Cas9-induced precise gene editing in mammalian cells. Nat. Biotechnol..

[100] Maruyama T., Dougan S.K., Truttmann M.C., Bilate A.M., Ingram J.R., Ploegh H.L. (2015). Increasing the efficiency of precise genome editing with CRISPR-Cas9 by inhibition of nonhomologous end joining. Nat. Biotechnol..

[101] Cox D.B., Platt R.J., Zhang F. (2015). Therapeutic genome editing: Prospects and challenges. Nat. Med..

[102] Klug A. (2010). The discovery of zinc fingers and their applications in gene regulation and genome manipulation. Annu. Rev. Biochem..

[103] Alwin S., Gere M.B., Guhl E., Effertz K., Barbas C.F., Segal D.J., Weitzman M.D., Cathomen T. (2005). Custom zinc-finger nucleases for use in human cells. Mol. Ther..

[104] Zhang C., Xu K., Hu L., Wang L., Zhang T., Ren C., Zhang Z. (2015). A suicidal zinc finger nuclease expression coupled with a surrogate reporter for efficient genome engineering. Biotechnol. Lett..

[105] Townsend J.A., Wright D.A., Winfrey R.J., Fu F., Maeder M.L., Joung J.K., Voytas D.F. (2009). High-frequency modification of plant genes using engineered zinc-finger nucleases. Nature.

[106] Osakabe Y., Osakabe K. (2015). Genome editing with engineered nucleases in plants. Plant Cell Physiol..

[107] Guan G., Zhang X., Naruse K., Nagahama Y., Hong Y. (2014). Gene replacement by zinc finger nucleases in medaka embryos. Mar. Biotechnol..

[108] Menke D.B. (2013). Engineering subtle targeted mutations into the mouse genome. Genesis.

[109] Zimmerman K.A., Fischer K.P., Joyce M.A., Tyrrell D.L. (2008). Zinc finger proteins designed to specifically target duck hepatitis B virus covalently closed circular DNA inhibit viral transcription in tissue culture. J. Virol..

[110] Lee H.J., Kim E., Kim J.S. (2010). Targeted chromosomal deletions in human cells using zinc finger nucleases. Genome Res..

[111] Lee H.J., Kweon J., Kim E., Kim S., Kim J.S. (2012). Targeted chromosomal duplications and inversions in the human genome using zinc finger nucleases. Genome Res..

[112] Wang J., Friedman G., Doyon Y., Wang N.S., Li C.J., Miller J.C., Hua K.L., Yan J.J., Babiarz J.E., Gregory P.D. (2012). Targeted gene addition to a predetermined site in the human genome using a ZFN-based nicking enzyme. Genome Res..

[113] Kandavelou K., Mani M., Durai S., Chandrasegaran S. (2005). “Magic” scissors for genome surgery. Nat. Biotechnol..

[114] Urnov F.D., Miller J.C., Lee Y.L., Beausejour C.M., Rock J.M., Augustus S., Jamieson A.C., Porteus M.H., Gregory P.D., Holmes M.C. (2005). Highly efficient endogenous human gene correction using designed zinc-finger nucleases. Nature.

[115] Boch J., Scholze H., Schornack S., Landgraf A., Hahn S., Kay S., Lahaye T., Nickstadt A., Bonas U. (2009). Breaking the code of DNA binding specificity of TAL-type III effectors. Science.

[116] Sun N., Zhao H. (2013). Transcription activator-like effector nucleases (TALENs): A highly efficient and versatile tool for genome editing. Biotechnol. Bioeng..

[117] Joung J.K., Sander J.D. (2013). TALENs: A widely applicable technology for targeted genome editing. Nat. Rev. Mol. Cell Biol..

[118] Lamb B.M., Mercer A.C., Barbas C.F. (2013). Directed evolution of the TALE N-terminal domain for recognition of all 5′ bases. Nucleic Acids Res..

[119] Ain Q.U., Chung J.Y., Kim Y.H. (2015). Current and future delivery systems for engineered nucleases: ZFN, TALEN and RGEN. J. Control. Release.

[120] Groner A.C., Meylan S., Ciuffi A., Zangger N., Ambrosini G., Denervaud N., Bucher P., Trono D. (2010). KRAB-zinc finger proteins and KAP1 can mediate long-range transcriptional repression through heterochromatin spreading. PLoS Genet..

[121] Sripathy S.P., Stevens J., Schultz D.C. (2006). The KAP1 corepressor functions to coordinate the assembly of *de novo* HP1-demarcated microenvironments of heterochromatin required for KRAB zinc finger protein-mediated transcriptional repression. Mol. Cell. Biol..

[122] Hsu P.D., Lander E.S., Zhang F. (2014). Development and applications of CRISPR-Cas9 for genome engineering. Cell.

[123] Sander J.D., Joung J.K. (2014). CRISPR-Cas systems for editing, regulating and targeting genomes. Nat. Biotechnol..

[124] Bondy-Denomy J., Davidson A.R. (2014). To acquire or resist: The complex biological effects of CRISPR-Cas systems. Trends Microbiol..

[125] Fineran P.C., Dy R.L. (2014). Gene regulation by engineered CRISPR-Cas systems. Curr. Opin. Microbiol..

[126] Kew M.C. (2011). Hepatitis B virus X protein in the pathogenesis of hepatitis B virus-induced hepatocellular carcinoma. J. Gastroenterol. Hepatol..

[127] Weber N.D., Stone D., Sedlak R.H., de Silva Feelixge H.S., Roychoudhury P., Schiffer J.T., Aubert M., Jerome K.R. (2014). AAV-mediated delivery of zinc finger nucleases targeting hepatitis B virus inhibits active replication. PLoS ONE.

[128] Chen J., Zhang W., Lin J., Wang F., Wu M., Chen C., Zheng Y., Peng X., Li J., Yuan Z. (2014). An efficient antiviral strategy for targeting hepatitis B virus genome using transcription activator-like effector nucleases. Mol. Ther..

[129] Lin S.R., Yang H.C., Kuo Y.T., Liu C.J., Yang T.Y., Sung K.C., Lin Y.Y., Wang H.Y., Wang C.C., Shen Y.C. (2014). The CRISPR-cas9 system facilitates clearance of the intrahepatic HBV templates *in vivo*. Mol. Ther. Nucleic Acids.

[130] Seeger C., Sohn J.A. (2014). Targeting hepatitis B virus with CRISPR-cas9. Mol. Ther. Nucleic Acids.

[131] Dong C., Qu L., Wang H., Wei L., Dong Y., Xiong S. (2015). Targeting hepatitis B virus cccDNA by CRISPR-cas9 nuclease efficiently inhibits viral replication. Antivir. Res..

[132] Qi Z., Li G., Hu H., Yang C., Zhang X., Leng Q., Xie Y., Yu D., Zhang X., Gao Y. (2014). Recombinant covalently closed circular hepatitis B virus DNA induces prolonged viral persistence in immunocompetent mice. J. Virol..

[133] Kinetics of Knockdown from RNAi Therapeutic ARC-520 on HBV RNA, DNA and Antigens in Mice and Chimpanzees. http://www.arrowheadresearch.com/sites/default/files/MolBiol_HBV_poster_102313.pdf.

[134] Yuan T.H., Li M.Y., Li W.Y., Li H., Jiang Z.H. (2007). Combinatorial effects of ST6Gal I siRNA and antisense oligonucleotide-mediated gene silence on metastasis ability of cervical carcinoma cells. Sichuan Da Xue Xue Bao Yi Xue Ban.

